# Understanding Digital Literacy of Persons With Dementia and Their Caregivers: A Scoping Review and an Evolutionary Concept Analysis of Empirical Studies

**DOI:** 10.1177/07334648251348703

**Published:** 2025-06-19

**Authors:** Hannah Cho, Emma Cho, Sang Bin You, Justine S. Sefcik, Nancy A. Hodgson, George Demiris

**Affiliations:** 1 6572 University of PennsylvaniaSchool of Nursing, Philadelphia, PA, USA; 2 15775 Drexel University College of Nursing and Health Professionals, Philadelphia, PA, USA

**Keywords:** digital literacy, dementia, caregiving

## Abstract

This study constitutes both a scoping review and a concept analysis, as it systematically examines digital literacy across settings (home, assisted living facilities, and other long-term care facilities) in the context of persons with dementia and their caregivers. We performed a scoping review of 22 empirical studies to examine how the literature has defined digital literacy in dementia care, followed by a concept analysis to conceptualize digital literacy’s antecedents, attributes, and consequences. Our analysis revealed three antecedents of digital literacy: technology-enhanced support systems, individual characteristics, and sociodemographic characteristics, which collectively impact persons with dementia and their caregivers’ abilities to utilize digital resources effectively. We identified two critical attributes—technological skills and critical thinking skills—to evaluate digital health information. Our findings revealed that digital literacy is tied to several important consequences: technology acceptance, impacts on psychosocial factors, and the promotion of digital equity. These findings provide a foundation for future research that can incorporate the core components of digital literacy when designing technology-mediated interventions. While our findings conceptualized digital literacy among persons living with dementia and their caregivers, more research is needed to promote standardized terminology and consider the digital literacy level of end-users in developing technology-mediated interventions in the future.

**What this paper adds**

• This manuscript identifies key antecedents, attributes, and consequences related to digital literacy among persons with dementia and their caregivers.
• It provides a conceptual framework with a better understanding of how digital literacy affects and is affected by various aspects of caregiving, including technical skills, information access, and communication abilities.

**Applications of study findings**

• Our conceptualized definition and framework of digital literacy can inform policy and practice, helping to standardize terminology and inform the design of digital tools for this vulnerable population.
• Our review identifies gaps in the current understanding of digital literacy among caregivers and suggests areas where future research could help develop more effective support strategies.

## Introduction

The rapid evolution of digital health shows a pivotal shift in how healthcare is delivered, accessed, and experienced. This transformation has been driven by current public health challenges, such as the demands of aging populations, escalating healthcare costs, and persistent disparities in access to care (Abernethy et al., 2022; Daniels & Bonnechère, 2024). In recent decades, digital health technologies—encompassing telemedicine, electronic health (eHealth) systems, mobile health (mHealth) applications, remote patient monitoring devices, and generative artificial intelligence—have gained significant attention due to their vast potential to revolutionize healthcare delivery (Stoumpos et al., 2023). By leveraging computing platforms, connectivity, software, and sensors, such technologies could improve accessibility to medical services, particularly by addressing various challenges in community settings, including assisted living facilities and nursing homes.

As digital literacy continues to evolve with advancements in technology, understanding its clear definition becomes increasingly important. Digital literacy is not solely about mastering specific technologies, such as smartphones, tablets, or laptops, but rather encompasses a person’s ability to adapt to new technologies and navigate the changing digital landscape effectively. The adaptability highlights the dynamic nature of digital literacy, which evolves in response to technological advancements and the changing digital landscape (Reddy et al., 2020, 2023). However, the concept of digital literacy remains heterogeneous across disciplines, with varying definitions depending on the context. For example, a health literature review revealed significant variability in how the term “digital literacy” is used, underscoring the need for a comprehensive understanding of its diverse aspects. Such an understanding could assist researchers and engineers in designing technologies that accommodate older adults’ cognitive abilities and help them adapt to evolving digital landscapes (Kang et al., 2023).

Persons with dementia often face unique challenges in adapting to technologies due to cognitive decline, sensory changes, and limited digital literacy (Bertolazzi et al., 2024; Wilson et al., 2022). While alarms and reminders can support memory, barriers such as insufficient accommodations for motor and sensory impairments often hinder their use (Wilson et al., 2022). Technologies designed to assist with cognitive function or daily tasks are rarely tailored for this population (Joddrell & Astell, 2016; Lazar et al., 2017; Leung et al., 2022). Caregivers of persons with dementia also face significant challenges with digital literacy, especially as they age and struggle to adapt to evolving technologies (Samari et al., 2024; Sixsmith et al., 2022). Supporting caregivers’ unique needs is crucial for their well-being and ensuring high-quality care for persons with dementia. Previous research has primarily focused on examining technology usability and barriers directly, rather than analyzing the underlying concepts of digital literacy in dementia care. This approach has created a gap in addressing the specific needs of persons with dementia (Chien et al., 2024; Holthe et al., 2018).

A clear understanding of digital literacy is essential for investigating the complexities of digital technology and its role in technology-mediated interventions. However, a comprehensive review or concept analysis focusing exclusively on digital literacy among persons with dementia and their caregivers has yet to be conducted. Therefore, this paper aims to clarify the concept of digital literacy through a scoping review and Rodgers’ evolutionary concept analysis method (Rodgers & Knafl, 1993).

## Methods

### Designs

This study combines a scoping review and a concept analysis to systematically examine the theoretical underpinnings and operational definitions of technology across various community settings, including home, assisted living facilities, and other long-term care facilities. It comprehensively reviews existing evidence regarding its applications, effectiveness, and limitations, without being confined to a singular evaluation methodology (Lam Wai Shun et al., 2022).

### Stages of a Scoping Review

For this scoping review, we followed the framework by Arksey and O'Malley (2005): identifying the research question, selecting relevant studies, charting the data, and summarizing and reporting the results (Arksey & O'Malley, 2005).

#### Eligibility Criteria and Search Strategy

We performed a scoping review using the PRISMA (the Preferred Reporting Items for Systematic Reviews and Meta-Analyses) guidelines and checklists to structure our research process and reporting (Page et al., 2021). A search was completed in August 2024 using PubMed, Embase, CINAHL, PsychINFO, and Scopus. See Table 1 for a summary of search terms. This search was completed with the assistance of an experienced librarian. The inclusion criteria are as follows: studies must focus on persons with dementia or their caregivers in community settings, examine technology-mediated interventions or technology-related applications for caregiving, and report outcomes or discuss digital literacy, including skill development, barriers, and facilitators of technology use. Only empirical studies using quantitative, qualitative, or mixed methods are included. We excluded any commentary, reviews, and protocol papers that are not empirical studies.

**Table 1.** Search Strategy.

Summary of Database (DB) Search Terms	
DB	Keywords
PubMed	(“Dementia”[MeSH Terms] OR Dementia [tiab] OR “Alzheimer’s diseases”[tiab] OR “mild cognitive impairment”[tiab]) AND(“Computer Literacy”[MeSH Terms] OR “ehealth literacy” [tiab] OR “mhealth literacy” [tiab] OR “computer literacy” [tiab] OR “digital literacy” [tiab] or “technology literacy”[tiab] OR “digital divide”[tiab] OR “technology divide”[tiab]) AND (“Caregivers”[MeSH Terms] OR “caregivers”[all fields] OR “caregiving”[all fields]) AND (“Aged” [MeSH Terms] OR “aged” [tiab] OR “older adults” [tiab])
CINAHL	((MH “Dementia+” OR MH “Alzheimer’s Disease” OR MH “Mild Cognitive Impairment” OR “cognitive impairment”)) AND ( (MH “Computer Literacy” OR “digital literacy” OR “technology literacy” OR “computer literacy” OR “digital divide” OR “ehealth literacy” OR “mhealth literacy” OR “technology divide”)) AND ((MH “Caregivers” OR caregivers OR family OR “significant others”)) AND ((MH “Aged, 80 and Over+” OR “older adults”))
Embase	‘computer literacy’/exp OR ‘digital literacy’ OR ‘digital divide’ OR ‘technology literacy’ OR ‘ehealth literacy’ OR ‘technology divide’ OR ‘mhealth literacy’
	AND ‘dementia’/exp OR ‘alzheimer disease’ OR ‘mild cognitive impairment’
	AND ‘caregiver’/exp OR ‘family’ OR ‘carer’ AND ‘aged’/exp
Scopus	(dementia OR alzheimers OR “Alzheimer’s Disease” OR “mild cognitive impairment”) AND (“older adults” OR seniors OR aged) AND (“computer literacy” OR “digital literacy” OR “e-health literacy” OR “mhealth literacy” OR “technology literacy” OR “digital divide”) AND (caregivers OR family OR “significant others”)
PsychInfo	(mild cognitive impairment OR dementia) AND (older adults OR aging) AND (digital divide OR literacy) AND (caregivers OR family) [All Abstracts and Summary]

#### Data Analysis

We organized selected studies using a matrix. This structured analyzing matrix was used to distinguish research fields, research objectives, research designs, inclusion of concepts, and keywords. We used the software Covidence to conduct a comprehensive review of empirical studies. To ensure reliability, all data was extracted by more than one author, with their results compared. After the individual analyses were concluded, cross-checking by two authors confirmed the inclusion of all 22 studies in the analysis. The final 22 studies were independently extracted by three authors (HC, EC, SBY) and cross-checked by at least two authors for validity. In conducting content analysis (Elo & Kyngäs, 2008), line-by-line coding was used to extract codes and group data into categories, with all data organized in an Excel file. We created the Table of Evidence by systematically extracting and organizing data from relevant studies, using columns such as Author (Publication Year), Study Designs, Setting, Participants, Objectives, Measurements, Key Findings, and Limitations to ensure comprehensive analysis and comparison of evidence, particularly in relation to digital literacy.

#### Study Risk of Bias Assessment

Three authors (HC, EC, SBY) independently assessed the quality of 22 articles using the Mixed Methods Appraisal Tool (Hong et al., 2018). The tool consists of a five-item checklist for each study type (qualitative study, randomized controlled trial, non-randomized trials, quantitative descriptive study, and mixed methods), with response options being “Yes,” “No,” and “Unknown.” The tool does not provide a pre-defined cut-off score to classify studies as “high” or “low” quality.

Thus, we are also reporting based on their total number of “yes” responses in the studies and interpreting what aspects were most frequently reported or not. We also decided not to exclude any studies based on the quality assessment results. The evaluation results of each author were compared and discussed within the group to reconcile the differences.

### Stages of Concept Analysis

Following our scoping review, we identified a gap in understanding the concept of digital literacy and its evolution. We adopted Rogers’ evolutionary concept analysis (Rodgers & Knafl, 1993) to systematically explore digital literacy among persons living with dementia and their caregivers.

The six stages of this process include: (1) identifying the concept, (2) identifying and selecting appropriate setting and sample for data collection, (3) collecting data to identify the attributes of the concept and the contextual basis of the concept, (4) analyzing data regarding the above characteristics of the concept, (5) identifying the exemplar, and (6) identifying implications for further development of digital literacy.

## Results

### Study Selection

**Figure 1.**
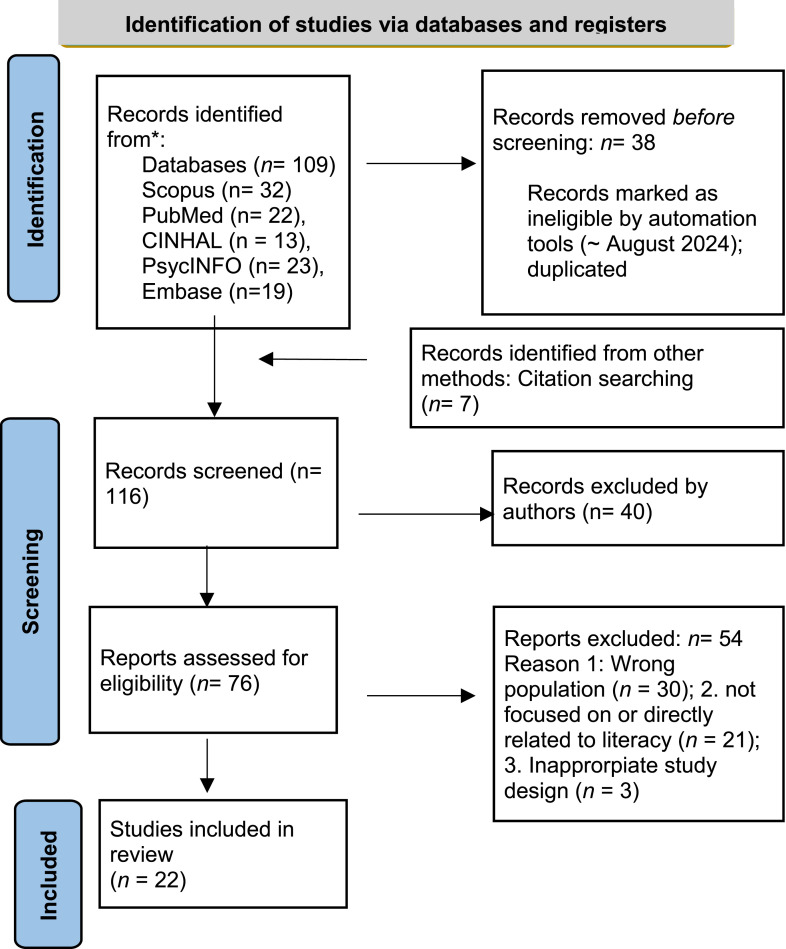
The preferred reporting items for systematic reviews and meta-analyses 2020.

Our initial search yielded a total of 109 studies, with an additional 7 studies identified through reference list reviews, bringing the overall total to 116 studies. After removing duplicates, three authors (HC, EC, SBY) independently screened the remaining 76 studies by title and abstract using the inclusion criteria and then cross-checked each other’s screenings. Any discrepancies between the three authors were discussed until a consensus was reached. If disagreement remained, the last author (GD) served as a tiebreaker. After excluding 40 studies from the abstract reviews, the three authors reviewed full texts of the remaining studies and further excluded 51 studies that did not meet the inclusion criteria. When discrepancies among the authors occurred, authors discussed and reconciled the discrepancies. Of the 54 studies excluded, 30 studies were irrelevant populations such as pediatrics and cancer patients. Twenty-one studies were not focused on or directly related to digital literacy, and three had ineligible study design approaches such as reviews or disseminations of the study protocol. (See Figure 1).

### Study Characteristics

A total of 22 studies were included in this study. The publication years of the included studies ranged from 2016 to 2024. Most studies employed qualitative descriptive designs (N = 11), followed by quantitative descriptive (N = 6), mixed-methods (N = 3), one quantitative randomized controlled trial (Thompson et al., 2024), one quantitative non-randomized controlled trial (Efthymiou et al., 2022).

Sample sizes across studies varied significantly, from 7 to 304 participants. Average sample sizes ranged between 21 and 86. Participant demographics primarily included caregivers of persons with dementia (N = 14), followed by caregiver-persons with dementia dyads (N = 5), and three studies focused solely on persons with dementia.

The studies explored a variety of technologies relevant to dementia care, including eHealth modalities used (N = 5), telehealth and videoconferencing (N = 5), passive and active devices (N = 3), mobile health (N = 2), virtual cognitive stimulation therapy (N = 2), assistive devices (N = 2), patient portal websites (N = 2), and socially assistive robot (Arthanat et al., 2020).

### Risk of Bias in Studies

Table 2 summarizes the quality appraisal of the included studies. All 11 qualitative studies met the five criteria for qualitative descriptive studies. Among the six quantitative non-descriptive studies, most scored “yes” on questions 1, 2, and 4 but failed to demonstrate sample representation. Three mixed-methods studies lacked adequate integration of qualitative and quantitative components (Duggleby et al., 2019; Fisher et al., 2024; McLoughlin et al., 2023). One quantitative non-randomized controlled trial (Efthymiou et al., 2022) and one quantitative randomized controlled trial (Thompson et al., 2024) met most criteria but fell short on intervention adherence and administration.

**Table 2.** Mixed-Methods Appraisal Tool Results for the Included Studies.

Criteria	Yes	No	Unknown
Quality appraisal results of qualitative study (N = 11)			
1. Is the qualitative approach appropriate to answer the research question?	11	0	0
2. Are the qualitative data collection methods adequate to address the research question?	11	0	0
3. Are the findings adequately derived from the data?	11	0	0
4. Is the interpretation of results sufficiently substantiated by data?	11	0	0
5. Is there coherence between qualitative data sources, collection, analysis, and interpretation?	11	0	0
Quality appraisal results of quantitative descriptive study (N = 6)			
1. Is the sampling strategy relevant to address the research question?	6	0	0
2. Is the sample representative of the target population?	2	4	0
3. Are the measurements appropriate?	5	1	0
4. Is the risk of nonresponse bias low?	0	0	6
5. Is the statistical analysis appropriate to answer the research question?	5	1	0
Quality appraisal results of mixed methods study (N = 3)			
1. Is there an adequate rationale for using a mixed methods design to address the research question?	2	0	1
2. Are the different components of the study effectively integrated to answer the research question?	2	0	1
3. Are the outputs of the integration of qualitative and quantitative components adequately interpreted?	1	1	1
4. Are divergences and inconsistencies between quantitative and qualitative results adequately addressed?	1	1	1
5. Do the different components of the study adhere to the quality criteria of each tradition of the methods involved?	1	1	1
Quality appraisal results of non-randomized control trials (N = 1)			
1. Are the participants representative of the target population?	1	0	0
2. Are measurements appropriate regarding both the outcome and intervention (or exposure)?	1	0	0
3. Are there complete outcome data?	1	0	0
4. Are the confounders accounted for in the design and analysis?	1	0	0
5. During the study period, is the intervention administered (or exposure occurred) as intended?	0	0	1
Quality appraisal results of randomized control trials (N = 1)			
1. Is randomization appropriately performed?	1	0	0
2. Are the groups comparable at baseline?	1	0	0
3. Are there complete outcome data?	1	0	0
4. Are outcome assessors blinded to the intervention provided?	1	0	0
5. Did the participants adhere to the assigned intervention?	0	0	1

**Table 3.** Definition of the Surrogate Terms.

Term	Definition
Digital literacy (broad)	Technological skills and critical thinking skills to evaluate digital information
eHealth literacy	Individual’s ability to use a web-based system to seek health information comfortably and confidently
Technology literacy	Individual’s knowledge in how to identify and operate available technologies
Computer literacy	Individual’s ability to use computers and related technology

**Table 4.** Antecedents, Attributes, and Consequences With Empirical References.

Components of Concept	
Antecedents	Empirical References (study numbers)
Tech-enhanced support system	
Formal support	Caregiver training (14)
	Clinicians (18)
	IT support person (3)
	Help in setting up remote meetings (4)
Informal support	Live with younger generation family members to assist them (2)
	Caregivers’ technical support (10)
Digital environment	Internet connections (3,6, 22)
	Access to suitable technology and high-speed network (10, 12)
	User friendly digital system (17)
	Interacts with the technology in surrounding environment
	Types of information and communications technology (12)
Individual characteristics	
Health status	Cognition (5,12), caregiver burden (22)
Health literacy	Health literacy (8,13)
Tech familiarity and functional benefits	Familiar with technology (1,3,4,14,15)
	Necessary computer skills (3,7,11,15,16)
	Perceived benefits (15)
Caregiver burden	(22)
Sociodemographic characteristics	
Formal education	(8,15,19)
Income	(15,19)
Gender	(15,19)
Age	(1,18,21)
**Attributes**	**Empirical references**
Technological skills	Adapted to using various technologies, including videoconferencing (14)
	Know how to use and manipulate different types of digital devices (4)
	Ability to efficiently use computers and related technologies (6)
	Demonstrate knowledge and the ability to use surrounding technologies and make decisions about whether to engage with devices to seek support
Critical thinking skills to evaluate digital health information	Comprehension of knowledge and information related to digital health (13)
	The eHealth literacy survey (eHEALS) has two components: Information seeking and evaluation (8)
**Consequences**	**Empirical references**
Technology acceptance	More use of technology to access healthcare (4,7)
	Acceptability to online patient portal (4,9)
	Willingness to use technology in the future (3)
Impact on psychosocial factors	Negative: Technology anxiety (2), frustration (6), fear or mistrust (11)
	Positive: Confidence (14), social engagement (22)
Promotion of digital equity	Digital divide (2,11)

*Note.* 1. Arighi et al. (2021); 2. Arthanat et al. (2020); 3. Banbury et al. (2019); 4. Chirico et al. (2022); 5. Daly-Lynn et al. (2023); 6. Duggleby et al. (2019); 7. Dupont et al. (2023); 8. Efthymiou et al. (2022); 9. Engelsma et al. (2022); 10. Fisher et al. (2024); 11. Hicks et al. (2023); 12. Jakobsson et al. (2022); 13. Kotwal et al. (2021); 14. McLoughlin et al. (2023); 15. Oh et al. (2023); 16. Peri & Cheung (2023); 17. Ruggiano et al. (2019); 18. Smith et al. (2022); 19. Thompson et al. (2024); 20. Wang et al. (2021); 21. Wilding et al. (2021); 22. Yin et al. (2024).

**Table 5.** Summary of Included Studies.

Author (publication year)	Study designs	Setting, participants	Objectives	Measurements	Key findings	Limitations	Related to digital literacy
Arighi et al. (2021)	Quantitative descriptive	Italy, 108 patients with cognitive impairment	To describe the digital divide of a population of patients with dementia contacted by telemedicine during Italian lockdown for COVID-19 pandemic	Mini-mental status exam, whether patients were able to connect with neurologist on Microsoft Teams	Seventy-four patients connected with neurologist (successful televisit, 68.5%) and 34 patients were not able to perform televisit and were contacted by phone (failed televisit, 31.5%). No significant differences were observed among the two groups concerning age, gender, and education, but the prevalence of successful televisit was higher in the presence of younger caregivers	Small number of participants and the lack of accurate sociodemographic and socioeconomic information of caregivers	Digital divide is any uneven distribution in information and communications technologies between people
Arthanat et al. (2020)	Qualitative descriptive	USA, Caregivers of ADRD (N = 24)	To beta-test a novel socially assistive robot (SAR) with a cohort of ADRD caregivers and gather their perspectives on its potential integration in the home context	Guiding interview questions were derived from the UTAUT.	Adoption of the SAR, as an identified theme, was subject to the SAR’s navigability, care recipient engagement, adaptability, humanoid features, and interface design. In contrast, barriers leading to potential rejection were technological complexity, system failure, exasperation of burden, and failure to address digital divide	Both sources of data, the focus group and follow-up interviews, were derived from a single cohort of caregivers	Describing digital divide as a risk of rejection on socially assisted robot adoption (SAR)
Banbury et al. (2019)	Qualitative descriptive	Australia, 69 dementia caregivers (mean age 63 years; SD = 13.54)	To explore the use of technology and its perceived effects across different settings and countries	Three sub-scales from the e-Health Literacy Questionnaire (eHLQ): “Using technology to process health information,” “ability to actively engage with digital services,” and “motivated to engage with digital services”	Providing peer-support groups using telehealth may have the potential to develop self-sustaining peer networks for isolated caregivers of people with dementia	Small sample size and potential bias in recruiting participants who were digitally proficient	None
Chirico et al. (2022)	Qualitative descriptive	The UK, Italy, Australia and Poland 127 informal carers (mostly female, n = 98) and 15 people with dementia (mostly female, mean age = 69)	To explore the use of technology and its perceived effects across different settings and countries	Interview questions not available	Technology kept us alive during COVID-19; remote care was anything but easy; perceived technology limitations	People with dementia were underrepresented compared to an adequate number of carers	Digital literacy is familiar with technology, know how to use or manipulate digital devices
Daly-Lynn et al. (2023)	Qualitative descriptive	The UK. 22 people with dementia (20 female, 2 male) and 31 formal caregiver survey	To examine experiences of people with dementia and live in technology enriched supported care models	Survey and interviews (questions were focused on the general experience of living in a supported living environment and the technology within the environment	A lack of awareness about living alongside technology	A single country in the United Kingdom	To use the system when they need to
Duggleby et al. (2019)	Mixed methods	Canada; 199 carers of persons with ADRD	To (1) examine differences at three months in the outcomes of hope, self-efficacy, and health-related quality of life (HRQOL) scores in users (i.e., those who used MT4C at least once during the three-month period) compared with nonusers and (2) identify reasons for nonuse	Interviews, Survey: Hope (Herth Hope Index; HHI), self-efficacy (General Self-Efficacy Scale; GSES), and HRQOL (ShortForm 12-item health survey version 2; SF-12v2)	Users had significantly higher GSES scores than nonusers (*p* = .048). Reasons for nonuse of MT4C included the following: Caregiving demands, problems accessing MT4C (poor connectivity, computer literacy, and navigation of MT4C), and preferences (for paper format or face-to-face interaction). Problems accessing MT4C were related to poor internet connections, computer literacy, and difficulties navigating the site	Nature of a secondary analysis, low computer literacy and poor connectivity were not considered as exclusion criteria for the study, possible bias	Computer literacy is navigating
Dupont et al. (2023)	Qualitative descriptive	Belgium; 18 family caregivers of persons with dementia and 17 healthcare professionals	To define the content of an interactive website for persons with dementia and their family caregivers to support them in ACP and to assess the barriers and facilitators for potential users in finding and using such a website from the perspective of family caregivers and healthcare professionals	Interviews: Preferences regarding functionalities—how the content is delivered (e.g., video), options for a larger font, text-to-speech option, etc.—and the possible barriers and facilitators to finding and using the website were assessed using open questions. Moreover, we also asked about the need for separate sections within the ACP website for persons with dementia, family caregivers and dyads	Users had significantly higher General Self-Efficacy Scale scores than non-users. Web-based interventions, such as MT4C, have the potential to increase the self-efficacy of carers of persons with ADRD and MCC.	As a nature of secondary analysis, thus, follow-up interviews with nonuser participants were not conducted	Computer literacy refers to the ability to use computers and related technology efficiently
Efthymiou et al. (2022)	Quantitative non-randomized	Cyprus and Greece (174 primary informal carers of persons with dementia	To identify the levels of HL and eHL among carers of PwD in Greece and Cyprus and to search for the associations with other caring concepts	eHEALS and health literacy (HL) survey	Primary informal carers reported a high level of eHealth Literacy (eHL) and HL. Carers with higher HL were more likely to report higher score of eHL, caregiving self-efficacy and lower score of problematic/dysfunctional coping. A positive message was received with regard to the role of HL and eHL in the everyday caring	A difficult sample to recruit, could not easily approach the carers who did not attend training and awareness events or day services	N/A
Engelsma et al. (2022)	Qualitative (Delphi study)	The Netherlands: 37 ADRD experts (included 7∼9 informal caregivers) age range between 21–70	To prioritize these through a Delphi study with ADRD experts (case managers, informal caregivers, hospital healthcare professionals, district nurses, and researchers)	Total three rounds: 1st round-participant characteristics and potentially new insights into barriers to mHealth use for older adults living with ADRD; 2nd round—consensus questionnaire—barriers to mHealth use for this population (its impact and frequency); 3rd round—rejudged those barriers for which no consensus or minor consensus	Twenty-six barriers are considered to majorly affect mHealth use, most of which relate to cognition and frame of mind. This study contributes to the development of mHealth design guidelines that take into account the progressive and diverse ADRD- and aging-related symptoms negatively affecting mHealth implementation and adoption	Passive recruitment of participants for this study proved to be challenging; the participant group may not be fully representative for all ADRD experts	Computer literacy
Fisher et al. (2024)	Mixed methods	Brazil and India; 59 persons with mild to moderate dementia, supported by their family caregivers	To explore the feasibility and acceptability of online or virtual CST (vCST) delivery in India and Brazil, emphasizing barriers and facilitators to implementation	Interviews (intervention acceptability, feasibility, and experiences of implementation): Surveys (caregivers—the Zarit Burden Interview, dementia caregiver experience scale)	While online services broadened geographic access, challenges emerged concerning inadequate computer literacy, poor technology access, and establishing interpersonal connections online. Exploratory, uncontrolled analyses indicated positive trends in quality of life but negative trends in cognition and activities of daily living, but these results were not statistically significant	Not representative of the broader population of persons with dementia and their caregivers	Computer literacy
Hicks et al. (2023)	Qualitative descriptive	The UK; 42 caregivers of persons with dementia (20 co-resident and 22 non-co-resident carers)	To explore co-resident and non-co resident family carers of PLWD engaged with digital technologies during the pandemic	Interviews: (i) What were the participants’ experiences and perceptions of the pandemic currently, (ii) how did these compare to the start of the lockdown and what were their thoughts on how to move forward through the pandemic	Many of the carers engaged with information and communication technologies, and to a lesser extent assistive technologies, during the pandemic	All carers were already participating within the wider DETERMIND study and so represent a population that may be more inclined to undertake research	Technology literacy
Jakobsson et al. (2022)	Quantitative descriptive	Sweden; 32 participants (65–85 years old) with cognitive impairment of different origins	To compare how older adults with cognitive impairment perceive relevance and level of Everyday Information and Communication Technologies (EICT)	The Short Everyday Technology Use Questionnaire (S-ETUQ)	Interchangeably used with ICT literacy is using digital technology, communications tools and / or networks to access, manage, integrate, evaluate and create information in order to function in a knowledge society	Small sample size	None
Kotwal et al. (2021)	Quantitative descriptive study	USA; 20 dyads of caregivers and older adults with ADRD (mean age 70, SD = 9.0), 45% Spanish speaking, 60% limited health literacy	To determine the feasibility of the advance care planning program	Validated 15-item ACP Engagement Survey, self-rated overall health; one validated health literacy question (ie, confidence with forms); confidence using the internet	15% participant felt comfortable using the internet. Surrogate supporting the patient with facilitating technology. ACP engagement scores increased for 16 of 20 (80%) patients (*p* = .03) and 16 of 20 (80%) caregivers (*p* = .18). Caregivers experienced increased knowledge (3.8–4.7, *p* = .002) and self-efficacy (3.6–4.5, *p* = .034) for ACP	Small sample size	Technological knowledge on computer and navigating the website
McLoughlin et al. (2023)	Mixed methods	The UK, 39 carers of persons with dementia	To explore the experiences of carers of persons with dementia who participated in videoconferencing support groups during the COVID-19 pandemic to investigate their preferences and experiences with online, hybrid, and face-to-face support	Online questionnaire (demographic status, and the type of support group that they attended by asking what happened at a typical session) and interviews (to elicit a more in-depth discussion of the participant’s experiences with peer support groups, how the pandemic affected them as a carer, and what support they would like to receive in the future)	Themes (1) perceptions of online support groups and (2) preferences for future support	None	N/A
Oh et al. (2023)	Quantitative descriptive	USA; 204 family and unpaid caregivers of persons with ADRD	To investigate both chronic health conditions and the utilization of patient portals, focusing particularly on caregivers responsible for individuals with ADRD.	Survey (the Health Information National Trends Survey 2018–2020)	A significant proportion (46.6%) of ADRD caregivers had never accessed their patient portals. The limited utilization of patient portals among caregivers responsible for individuals with ADRD, particularly those with lower education, advanced age, and few chronic conditions, becomes apparent due to challenges associated with digital literacy and discomfort with computers	The cross-sectional nature of the data collected in the national database limits the ability to assess cause and effect, and only allows for investigation of relationships and the strength of those relationships	N/A
Peri & Cheung (2023)	Qualitative descriptive	New Zealand; 12 family carers of persons with dementia	To explore the roles and experiences of carers in accessing virtual CST	Interviews (capture information about the participant’s experience of supporting a persons with dementia to attend vCST during the COVID-19 pandemic, and to elicit suggestions on improving the implementation of vCST in a home environment)	Carers reported positive responses to vCST that provided their family member living with dementia with social contact and cognitive stimulation during lockdown	A small sample size	N/A
Ruggiano et al. (2019)	Qualitative descriptive	USA; 36 caregivers of persons with ADRD	To capture a more in-depth perspective on how ADRD caregivers perceive existing and potential of technology to improve ADRD caregiving and care	Interviews: (a) In what ways do you use technologies for caregiving? (b) How might a [clinical assessment tool, medication management tool, list of links and resources, alert system that sends messages to the provider] feature be helpful/not helpful for daily caregiving? (c) What kinds of caregiving tasks do you have challenges with that technology could potentially be of help?	Thematic findings suggest a conceptual model for designing ADRD caregiver technologies. The findings suggest that eHealth and individual technologies may not fully meet the needs of caregivers as they navigate the larger systems within which they provide care. Findings highlight the need to develop technologies for caregivers that are effective, easy to use, and more widely disseminated – especially for caregivers from disadvantaged backgrounds	A small sample size representative of the larger caregiver population; focus group interviews did not allow the research team to fully explore the research question with each individual participant in the study	Computer literacy—ease of use
Smith et al. (2022)	Qualitative descriptive	Canada; 30 stakeholders and 22 patient-family participant dyads	To explore: (1) receptivity to SHARING Choices; (2) perceived barriers or facilitators for implementing SHARING Choices; and (3) adaptations to support SHARING Choices implementation	Interviews: Receptivity to SHARING Choices components across CFIR domains	Enablers of SHARING choices included adaptability of the intervention, purposive engagement of family (particularly for persons with dementia), consistency with organizational priorities, and the relative advantage of SHARING Choices compared to current practices. Perceived barriers to implementation included intervention complexity, space constraints, workflow, and ACP hesitancy. The ACP facilitator was perceived as supportive in addressing individual and organizational implementation barriers including patient health and technology literacy and clinician time for ACP discussions	A small sample size; limited racial and ethnic diversity of the participants, small heterogenous group	N/A
Thompson et al. (2024)	Quantitative randomized controlled trial	USA; 163 family caregivers of persons with dementia	To evaluate the feasibility and usability of a technology-delivered intervention designed for family caregivers	Computer proficiency was measured by the Computer Proficiency Questionnaire short form (CPQ-12)	CPQ-12 scores can be used as a screening tool to identify those who may need additional support to engage with and benefit from technology-delivered interventions	This sample was highly educated and proficient with technology	Computer proficiency
Wang et al. (2021)	Quantitative non-randomized	China; 300 primary family caregiver- care recipient dyads	To examine the association between eHealth literacy, education, and caregiver burden among Chinese caregivers of older adults with cognitive impairment	Eight-item Chinese eHealth Literacy Scale	An interaction effect between eHealth literacy and education on caregiver burden was identified. eHealth literacy was positively associated with caregiver burden among caregivers with less than a high school education, but not among those with a high school education or above. eHealth literacy is salient in the burden experienced by caregivers with low education	Cross-sectional in nature	eHealth literacy
Wilding et al. (2021)	Qualitative descriptive	Australia; 39 participants (carers, volunteer, healthcare staffs of persons with dementia, average age was 66 years old) and mostly female (82%)	To increase access to information, support, and connection for carers of rural persons with dementia, via a co-designed, integrated website/mobile application (app) and Zoom videoconferencing	Qualitative data (memos, transcripts of focus groups and interviews)	The volunteers reported that the Verily Connect app was easy to use and they felt they derived benefit from volunteering. The volunteers had less volunteering work than they desired due to low numbers of carer participants; they reported that older rural carers were partly reluctant to join the trial because they eschewed using online technologies, which was the reason for involving volunteers from each local community	Small sample size	N/A
Yin et al. (2024)	Quantitative descriptive	USA; 140 caregivers of persons with dementia	Survey (eHeals)	To investigate the perceptions and utilization of online peer support through a survey	Our findings show that the behavior of accessing any online community was significantly associated with participants’ belief in the value of online peer support (*p* = .006)	Selection bias	eHealth literacy

*Note.* ADRD = Alzheimer’s diseases and related diseases; CST = cognitive stimulation therapy; N/A = not applicable.

### Results of Concept Analysis

#### Stage 1: Concept of Interest and Surrogate Terms

This study follows the steps outlined by Roger’s evolutionary concept analysis (Rodgers & Knafl, 1993), which started with identifying the concept of interest along with any related terms. The term *digital literacy* was rarely used specifically for the subject of interest, especially for persons with dementia. Alternate phrases used to convey the concept are referred to as surrogate terms (Rodgers & Knafl, 1993) for digital literacy, including eHealth literacy (Banbury et al., 2019; Efthymiou et al., 2022; Jakobsson et al., 2022; Ruggiano et al., 2019; Wang et al., 2021), computer literacy (Duggleby et al., 2019; Dupont et al., 2023; Engelsma et al., 2022; Thompson et al., 2024), and technology literacy (Hicks et al., 2023; Smith et al., 2022). Health literacy is the ability of individuals to find, understand, and use health information to make informed health decisions (National Institutes of Health, n.d.). It includes skills like reading comprehension, numeracy, and engaging with health resources, from medication labels to online portals (Efthymiou et al., 2022; Miller, 2016). Different from digital literacy, health literacy does not necessarily involve the ability to use any technology. Although distinct, health and digital literacy are becoming increasingly interdependent, as individuals need digital skills to access online health resources effectively, consequently affecting their health (Efthymiou et al., 2020, 2022).

All three surrogate terms, computer literacy, eHealth literacy, and technology literacy, overlap to some extent, especially as technology becomes increasingly integrated into everyday life and health management. Computer literacy is the foundational skill, providing the basic understanding of how to use computers (Duggleby et al., 2019; Dupont et al., 2023; Engelsma et al., 2022; Thompson et al., 2024). Technology literacy builds upon that by introducing a broader understanding of other technology tools, devices, and platforms (Hicks et al., 2023; Smith et al., 2022). E-health literacy, while being a more specialized domain, requires both computer and technology literacy to navigate and access digital health information (Banbury et al., 2019; Efthymiou et al., 2022; Jakobsson et al., 2022; Ruggiano et al., 2019; Wang et al., 2021). In this study, we refer to digital literacy as a comprehensive concept encompassing the other three surrogate terms (Table 3).

#### Stage 2: Identify the Setting and Sample for Data Collection

We examined persons with dementia and their caregivers in community settings. This setting is particularly relevant, as it allows us to examine the real-world interactions and challenges faced by this population within their daily environments rather than in acute hospital settings.

#### Stages 3 and 4: Identify and Analyze the Attributes, Antecedents, and Consequences of Digital Literacy

##### Antecedents

Antecedents are factors that precede a concept (Rodgers & Knafl, 1993). These antecedents are essential for understanding what drives or influences a specific behavior or event. In the context of digital literacy, antecedents could include factors that can influence how effectively a person will engage with or use digital technologies (Table 4 and 5).

##### Tech-Enhanced Support System

Included studies emphasized the significant roles of *tech-enhanced support systems* and *individual factors* in understanding digital literacy in persons with dementia and their caregivers. In this study, we conceptualize the digital environment as digital infrastructure and digital devices that communicate to manage content and activities. The digital environment encompasses digital infrastructure and platforms (Banbury et al., 2019; Daly-Lynn et al., 2023; Duggleby et al., 2019; Fisher et al., 2024; Jakobsson et al., 2022; Ruggiano et al., 2019), formal support through healthcare providers, technical assistance (Banbury et al., 2019; Chirico et al., 2022; McLoughlin et al., 2023; Smith et al., 2022), and informal support from family and community members (Arthanat et al., 2020; Fisher et al., 2024). Together, these factors constitute *the tech-enhanced support system*.

Technology-enhanced support system includes people with technical skills, and online resources. Studies (Arthanat et al., 2020; Banbury et al., 2019; Engelsma et al., 2022) underscored the importance of support from IT support staff, healthcare providers, and tech savvy family members for persons with dementia and their caregivers (Banbury et al., 2019; Fisher et al., 2024; Hicks et al., 2023; Smith et al., 2022). For instance, clinicians and nurses offered valuable insights into how accessing advanced care planning websites aligned with their healthcare practices and patient care (Smith et al., 2022). This formal support system helped to enhance persons with dementia and their caregiver’s overall digital literacy.

Studies have shown that when older adults, including those with dementia, receive help from younger, tech-savvy family members, they are more likely to overcome their initial reluctance toward or fear of digital devices (Arthanat et al., 2020). Members of younger generations are generally more familiar with technology and can offer practical, hands-on guidance that feels less intimidating for older adults. Moreover, the familiar and trusted environment provided by family members fosters a sense of reassurance, making it easier for older adults to navigate the complexities of digital tools without feeling overwhelmed (Fisher et al., 2024).

Many authors have emphasized the importance of internet connection quality as a crucial factor for digital literacy (Banbury et al., 2019; Duggleby et al., 2019). The quality of internet access and the availability of technology are interconnected factors that influence how easily persons with dementia and their caregivers can access digital tools and acquire the skills needed to navigate them. Similarly, individuals from marginalized or underserved communities may have less access to digital technologies (Fisher et al., 2024; Jakobsson et al., 2022) or internet connections (Fisher et al., 2024; Jakobsson et al., 2022; Yin et al., 2024) that impede their digital literacy. For instance, a study on the usability of virtual cognitive stimulation therapy among dementia caregivers in India and Brazil found that caregivers struggled with the intervention because of limited access to appropriate technology and computer literacy, further exacerbated by cognitive impairment (Fisher et al., 2024).

##### Individual Factors

*Individual factors,* defined as unique traits that describe individuals such as their physical traits, personality, and social circumstances, include technology familiarity and functional benefits (Arighi et al., 2021; Banbury et al., 2019; Chirico et al., 2022; McLoughlin et al., 2023; Oh et al., 2023), necessary computer skills (Banbury et al., 2019; Dupont et al., 2023; Hicks et al., 2023; Oh et al., 2023; Peri & Cheung, 2023), and perceived benefits (Oh et al., 2023).

In the context of health, especially that of persons with dementia, the authors emphasized the critical role of cognitive function as a precursor to digital literacy. Two studies discussed how cognitive abilities significantly impact an individual’s ability to use digital technologies, highlighting the need for customized approaches to improve digital literacy among persons with dementia. Daly-Lynn et al. (2023) noted that cognitive impairment can hinder individuals’ ability to remember how seek support when they need it. Another study (Jakobsson et al., 2022) emphasized that cognitive impairment can directly impact a person’s capacity to learn, remember, and apply new skills, including technology-related skills.

Additionally, familiarity with technology is crucial for persons with dementia and their caregivers (Arighi et al., 2021; Banbury et al., 2019; Chirico et al., 2022; McLoughlin et al., 2023; Oh et al., 2023), as it creates an environment conducive to developing digital literacy skills. When they are comfortable using technology, their ability to access information, communicate effectively, and manage health-related tasks while using digital health is enhanced.

Similarly, several studies have suggested that possessing essential computer skills—such as utilizing basic functions and performing simple tasks—is necessary for effective engagement with digital technologies (Banbury et al., 2019; Dupont et al., 2023; Hicks et al., 2023; Oh et al., 2023; Peri & Cheung, 2023). These skills facilitate the ability to navigate and access online platforms and empower individuals, particularly caregivers and persons with dementia. For example, one study used digital resources to establish a peer support group via telehealth for caregivers (Banbury et al., 2019). Such initiatives have the potential to create self-sustaining peer networks for isolated caregivers of persons with dementia. By utilizing technology, this method not only enables communication among caregivers but also promotes a sense of community and shared experience, ultimately improving their emotional well-being and support systems (Banbury et al., 2019). Health literacy is a key factor, as individuals with lower health literacy may have difficulty understanding the importance of using digital tools for health outcomes (Efthymiou et al., 2022; Kotwal et al., 2021). This lack of understanding can hinder their ability to access health information, use telehealth services, and engage with digital health applications effectively. There is also health literacy (Efthymiou et al., 2022; Kotwal et al., 2021), health status (Daly-Lynn et al., 2023; Jakobsson et al., 2022), and caregiver burden (Yin et al., 2024). Interestingly, caregiver burden, characterized by physical, emotional, and psychological strain, can limit caregivers’ ability to develop or utilize digital literacy skills (Yin et al., 2024). The demands of caregiving often leave little time or energy for seeking out digital resources, hindering their access to tools that could support their caregiving responsibilities.

##### Sociodemographic Characteristics

Sociodemographic characteristics include education (Efthymiou et al., 2022; Thompson et al., 2024), age (Arighi et al., 2021; Ruggiano et al., 2019; Wilding et al., 2021), gender (Oh et al., 2023; Thompson et al., 2024), income (Oh et al., 2023; Thompson et al., 2024), and education (Efthymiou et al., 2022; Oh et al., 2023; Thompson et al., 2024). For instance, one study highlighted the impact of education on digital literacy, particularly among caregivers of persons with dementia (Oh et al., 2023). Caregivers with lower educational levels demonstrated limited use of online patient portals, with 46.6% never accessing them. This suggests that education plays a critical role in shaping the digital literacy necessary to utilize online health resources effectively (Oh et al., 2023). Gender (Oh et al., 2023; Thompson et al., 2024) and age (Arighi et al., 2021; Ruggiano et al., 2019; Wilding et al., 2021) were also key individual factors for persons with dementia and their caregivers. Older adults often face greater challenges in adapting to new technologies. This is due to generational differences in exposure to and experience with them (Arighi et al., 2021; Ruggiano et al., 2019; Wilding et al., 2021).

#### Attributes

Attributes are the characteristics or qualities that describe an object, individual, or phenomenon. Attributes provide a deeper understanding of the components of digital literacy. These attributes can describe the skills and characteristics associated with a person with adequate digital literacy. Six studies identify key characteristics of digital literacy in the context of persons with dementia or their caregivers. These include *Technological skills* and *critical thinking skills to evaluate digital health information*, which are essential for managing the complexities of caregiving and health-related challenges (Chirico et al., 2022; Daly-Lynn et al., 2023; Duggleby et al., 2019; Efthymiou et al., 2022; Kotwal et al., 2021; McLoughlin et al., 2023).

##### Technological Skills

Some authors highlighted digital literacy as technological skills because these are the skills that individuals can use to effectively utilize available technologies and make informed decisions about when and how to engage with technologies. This includes operating various digital platforms, adapting to new technologies and navigating websites efficiently. Several authors alluded to the ability to use various technology and mobile applications and navigate websites (Chirico et al., 2022; Daly-Lynn et al., 2023; Duggleby et al., 2019; Kotwal et al., 2021). Technological skills empowered persons with dementia and their caregivers to access health-related information and operate various types of digital devices (Chirico et al., 2022). In healthcare, technology-mediated interventions, such as telehealth and online patient portals, allow individuals to access services remotely, improving their ability to manage their health information and communicate with providers (Chirico et al., 2022). Web-based interventions, such as My Tools 4 Care (MT4C) demonstrate a positive relationship between self-efficacy and the quality of life for family caregivers of persons with dementia and multiple chronic conditions (Duggleby et al., 2019). The authors emphasized the need for educational programs to enhance digital literacy and offering alternative methods for accessing MT4C beyond web-based platforms.

##### Critical Thinking Skills to Evaluate Digital Health Information

Digital literacy involves critical thinking and problem-solving skills. It requires persons with dementia and their caregivers to assess the reliability of online information, identify biases, and make informed decisions on the basis of digital health information (Efthymiou et al., 2022; Kotwal et al., 2021). This attribute fosters analytical skills that allow users to critically navigate the vast amounts of information available online. For example, Efthymiou et al. (2022) utilized the eHealth Literacy Survey (eHEALS) to assess the eHealth literacy levels for caregivers of persons with dementia. They found that caregivers with higher eHEALS scores, greater caregiving self-efficacy, and lower levels of problematic coping had better outcomes. The authors emphasized this attribute could enhance caregivers’ ability to navigate the overwhelming amounts of digital health information, facilitating informed decision-making based on credible and relevant data (Efthymiou et al., 2022).

#### Consequences

Consequences are the results, outcomes, or effects that follow from an action or behavior. They reflect the impact or implications of the behavior or phenomenon being studied. These can be the positive or negative effects resulting from an individual’s level of digital literacy. Understanding these components helps create a more comprehensive framework for explaining digital literacy within a specific context.

We analyzed three consequences of digital literacy as it is currently understood. First, *technology acceptance* involves more use of technology to access healthcare (Chirico et al., 2022; Dupont et al., 2023), acceptability to online patient portal (Chirico et al., 2022; Engelsma et al., 2022), and willingness to use technology in the future (Banbury et al., 2019). Second, *impact on psychological factors* is categorized into negative and positive feelings: negative feelings include technology anxiety (Arthanat et al., 2020), frustration (Duggleby et al., 2019), and fear or mistrust (Hicks et al., 2023); positive feelings include confidence (McLoughlin et al., 2023) and social engagement (Yin et al., 2024). Lastly, *the promotion of digital equity* is illustrated through studies highlighted the digital divide (Arthanat et al., 2020; Hicks et al., 2023).

##### Technology Acceptance

The willingness to adopt and integrate technology into daily routines is often influenced by an individual’s level of digital literacy. Several authors have emphasized the role of adequate digital literacy in increasing technology acceptance among caregivers (Banbury et al., 2019; Chirico et al., 2022; Dupont et al., 2023; Engelsma et al., 2022). Technology acceptance and digital literacy are related yet distinct concepts. While digital literacy can significantly influence technology acceptance, acceptance also depends on a person’s attitudes, perceptions, and willingness to adopt new technologies (Desmaryani et al., 2024). Technology acceptance models specifically address the psychological factors that affect adoption decisions, with technology acceptance frequently being linked to an individual’s level digital literacy (Granić & Marangunić, 2019; Nadal et al., 2020).

Most studies did not explicitly measure individuals’ digital literacy. Instead, they primarily focused on evaluating technology usability, such as web-based interventions (Dupont et al., 2023). Users such as family caregivers for persons with dementia often perceive digital tools as beneficial and are more comfortable navigating various digital technologies and online platforms. This comfort leads to increased use of advance care planning websites (Dupont et al., 2023). A single-group, multisite mixed methods study (Engelsma et al., 2022) involving 59 stakeholders found that caregivers with sufficient digital literacy were more likely to engage with online cognitive stimulation therapy. Conversely, those caregivers with inadequate digital literacy were hesitant to adopt new technology such as videoconferencing, because of a lack of comfort or technical support, resulting in missed opportunities to join the online peer support program throughout the dementia trajectory (Banbury et al., 2019).

##### Impacting Psychosocial Factors

Digital literacy also has implications for impacting psychosocial factors. Persons with dementia and caregivers who struggle with technology may experience feelings of frustration, fear, or even anxiety about technology (Arthanat et al., 2020; Duggleby et al., 2019; Hicks et al., 2023). The pressure to keep up with rapidly advancing technologies can cause psychological stress, particularly among older adults and their caregivers with limited social engagement. By contrast, improved digital literacy can foster a sense of empowerment, allowing individuals to engage more fully with digital resources and social networks (McLoughlin et al., 2023; Yin et al., 2024). For instance, Yin et al. (2024) found that among 172 dementia caregivers, those with higher digital literacy, as measured by the eHEALS scale, were more likely to access online communities. Their study revealed a significant relationship between caregivers’ belief in the value of online peer support and their participation in digital spaces (*p* = .006). On the other hand, a limited understanding of technologies can foster fear or mistrust toward technology, limiting its use. Hicks et al. (2023) found that caregivers of persons with dementia received phishing emails, which made them wary of online services, however, some overcame their fear and mastered the necessary digital skills, which gave them a sense of achievement but also enhanced social inclusion during the pandemic. These findings highlight how digital literacy enhances caregivers’ social engagement and their psychosocial factors.

##### Promotion of Digital Equity

A key consequence of promoting adequate digital literacy for all is the narrowing gap between individuals who have access to digital resources and those who do not. The promotion of digital equity is essential in addressing the digital disparities created by sociodemographic factors, such as income and age, which can significantly limit access to technology and opportunities for fostering digital literacy (Arthanat et al., 2020; Hicks et al., 2023).

Another study on Socially Assistive Robots found that caregivers recognized their potential to ease caregiving tasks. However, challenges like technological complexity and system failures, especially for those already facing digital inequalities, made it difficult for many to adopt the technology widely (Arthanat et al., 2020). The authors emphasized the importance of addressing the digital divide, especially for persons with dementia and their caregivers. Hicks et al. (2023) stressed the necessity of tailored services and interventions that can accommodate the preferences, needs, and technological skills of persons with dementia and their caregivers. These studies demonstrated that, regardless of their socio-demographic characteristics and cognitive impairments, persons with dementia and their caregivers should have the opportunity to thrive in a digital society.

#### Stage 5: Identifying an Exemplar of the Concept

**Figure 2.**
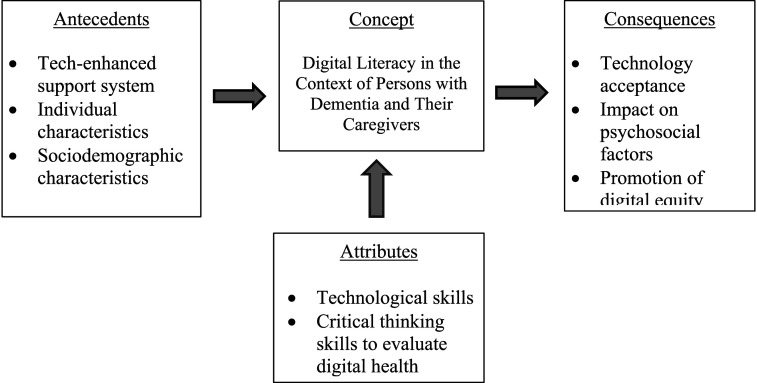
Proposed conceptual framework of digital literacy in the context of persons with dementia and caregivers.

Mary, a retired nurse caring for her husband with dementia, has varying attitudes toward technology. She is comfortable using her iPhone for basic tasks such as group chats and setting medication reminders (attributes), but she struggles with software updates and new features. Her tech-savvy son frequently assists her with these challenges (antecedents). However, she feels anxious about navigating online patient portals like MyChart, particularly when instructional videos are unavailable. She also feels uncertain about AI-based decision-support tools during clinical visits. Given that her husband is under hospice care, Mary lacks time to learn new digital tools, which complicates their integration into her daily routine. Feeling overwhelmed, she ultimately decides against using MyChart for doctor communication, despite recognizing its potential benefits (consequences). This example illustrates how an individual’s level of digital literacy directly impacts technology acceptance and usage in complex caregiving situations. See Figure 2 for the proposed conceptual framework of digital literacy in the context of persons with dementia and caregivers.

#### Stage 6: Identify Implications and Ideas for Further Development of Digital Literacy

Based on our findings on antecedents, attributes, and consequences, we also identified implications, hypotheses, and ideas for further development of digital literacy. We identified implications for various stakeholders, including clinicians, researchers, and policymakers. For clinicians, simplifying technology concepts and offering hands-on practice can enhance caregivers’ understanding and effective use of digital health resources. Breaking down complex digital concepts into manageable steps fosters a supportive environment for persons with dementia and their caregivers. Implications for researchers include designing and evaluating targeted digital literacy interventions that address the specific needs of dementia patients and caregivers and creating a foundation for practical, evidence-based programs that can be implemented in various settings. Researchers could also study the long-term effects of digital literacy on caregiver and patient well-being, examining how improved digital skills impact health outcomes, healthcare access, and caregiver-patient relationships. This finding could guide future interventions and policies. For policymakers, prioritizing digital literacy can significantly improve healthcare access for persons with dementia and their caregivers. Supporting digital navigator initiatives in resource-limited communities and incentivizing healthcare systems or community centers to provide digital literacy training—especially for using patient portals and online support networks—could significantly enhance the quality of care and support.

For further development, research should also explore ways to improve digital literacy in culturally diverse groups by offering materials in multiple languages and ensuring that the content is sensitive to cultural differences in caregiving practices. Finally, partnerships with healthcare providers to include digital literacy training in routine care settings could ensure that caregivers and persons with dementia are consistently supported in their digital engagement, allowing for more effective care management.

## Discussion

To our knowledge, our scoping review and concept analysis represents the first comprehensive effort to conceptualize “digital literacy” for persons with dementia and their caregivers through a rigorous scoping review and concept analysis. This definition—the ability to effectively use technology tools and critically evaluate digital health information—fills a critical gap in understanding how this vulnerable population navigates digital health environments.

We identified several antecedents (factors necessary for digital literacy development), including technology access, education, and previous experiences with technology. The consequences of digital literacy include improved access to information and enhanced ability to navigate digital environments effectively. These findings highlight the importance of digital literacy for persons with dementia and their caregivers in effectively accessing and utilizing digital health resources.

Our analysis identified only three studies focused solely on persons with dementia, while 14 studies examined digital literacy in caregivers of persons with dementia. The key challenges in addressing digital literacy for each group, as discussed earlier in this paper, are notably different. Despite inherent differences in cognitive function and needs, prior experiences and existing skills with technology may enable some persons with dementia to retain digital device uses, even as their cognitive functions decline with disease progression (Wu et al., 2019). These findings highlight that cognitive impairment alone should not lead to the marginalization of persons with dementia in future research or technology development. Excluding persons with dementia based solely on cognitive status could limit valuable insights into how persons with dementia interact with technology over time. Therefore, future studies and technological advancements should consider the existing capabilities and experiences of persons with dementia, ensuring they are included and represented in the design of inclusive, accessible digital health tools.

Additionally, the promotion of digital equity was identified as a significant outcome of digital literacy. By enhancing individuals’ digital skills and competencies, we can help bridge the existing digital divide, ensuring that everyone, regardless of their socioeconomic background, has equal access to technological resources and opportunities. This equity not only empowers individuals to engage fully in the digital world but also fosters greater inclusivity within communities (Yin et al., 2024). Increasing digital literacy enhances opportunities for collective social engagement and collaboration, fostering a more equitable society that leverages technology for the benefit of all.

Based on our findings, we recommend following several key factors to promote the successful adoption of digital health tools for persons with dementia and their caregivers. We grouped them into acceptability, usability, and adaptability. Acceptability can be fostered through engaging multiple stakeholders, offering tailored educational sessions, and providing accessible support personnel, such as IT staff or clinicians, to address specific needs. Usability is enhanced by designing user-friendly interfaces, utilizing multimodal content presentation and delivery adjustments, and minimizing screen time during training to reduce cognitive load. Finally, adaptability requires that training initiatives are community-based and incorporate hybrid formats. Customized courses for caregivers with low digital literacy, along with advanced staff training on device and data management, are also essential to ensure effective and sustainable integration of these digital tools.

### Strengths and Limitations

These findings not only contribute to the existing body of knowledge but also present a conceptual model of digital literacy specifically tailored to the context of persons with dementia and their caregivers. The model identifies key antecedents, attributes, and consequences of digital literacy, providing a more comprehensive understanding of how technology can be integrated into care for this population. An awareness of these elements is crucial for promoting the importance and practical application of digital literacy in care planning. Specifically, understanding the antecedents, attributes, and consequences of digital literacy enables healthcare providers and other stakeholders, such as researchers, to design more effective, accessible, and personalized interventions. This can enhance the quality of care by ensuring that both persons with dementia and their caregivers can effectively engage with digital tools, ultimately improving their access to healthcare resources and support systems.

This study has a few limitations. First, only studies published in English were included, potentially excluding valuable research published in other languages. These limitations may affect the comprehensiveness of our findings and suggest the need for further research that includes a broader range of studies and languages. Another limitation of this study is the potential for publication bias, as only peer-reviewed articles were included, which may overlook relevant gray literature, such as reports, theses, or non-peer-reviewed studies. This could limit the breadth of evidence considered in our analysis.

Future research should go beyond the current focus on the feasibility and effectiveness of digital technology interventions for persons with dementia and their caregivers. As dementia progresses, persons with dementia experience increasing vulnerability and more complex needs, making technology use challenging. Persons with dementia and their caregivers often lack the digital literacy and support needed to fully utilize these tools. Thus, future studies should also prioritize strategies to enhance digital literacy while continuing to evaluate telehealth usability and effectiveness. Researchers must also consider the broader social and psychological barriers faced by this population, incorporating these insights into interventions designed to enhance engagement and reduce disparities in digital health access. Thus, interventions aimed at improving digital literacy must address the complexity and interconnectedness of the attributes identified in this study.

## Conclusion

Based on our findings from this scoping review and concept analysis, we developed an operationalized definition of digital literacy and proposed a conceptual framework. Digital literacy refers to the technological skills needed to use digital technologies effectively and critical thinking skills to evaluate digital health information. Our proposed conceptual framework aims to guide future research and interventions focused on enhancing digital literacy among persons with dementia and their caregivers. By addressing the unique challenges, they face in managing health information and utilizing digital tools effectively, this concept of digital literacy and conceptual framework can inform health policy and clinical practice, ultimately improving support for this vulnerable population. These efforts are anticipated to facilitate increased technology acceptance and contribute to advancing digital health equity within this population.

## Supplemental Material

Supplemental Material - Understanding Digital Literacy of Persons With Dementia and Their Caregivers: A Scoping Review and an Evolutionary Concept Analysis of Empirical Studies Supplemental Material for Understanding Digital Literacy of Persons With Dementia and Their Caregivers: A Scoping Review and an Evolutionary Concept Analysis of Empirical Studies by Hannah Cho, Emma Cho, Sang Bin You, Justine S. Sefcik, Nancy A. Hodgson, and George Demiris in Journal of Applied Gerontology
