# Supplementary material for: Understanding Digital Literacy of Persons With Dementia and Their Caregivers: A Scoping Review and an Evolutionary Concept Analysis of Empirical Studies
Source: J Appl Gerontol. 2025 Jun 19;45(4):742–66. doi: 10.1177/07334648251348703 (PMC12989035; doi:10.1177/07334648251348703)
Supplement: Supplemental Material - Understanding Digital Literacy of Persons With Dementia and Their Caregivers: A Scoping Review and an Evolutionary Concept Analysis of Empirical Studies [file sj-pdf-1-jag-10.1177_07334648251348703.pdf]

Supplementary: Summary of Included Studies

Author (Publication Year)	Study Designs	Setting, Participants	Objectives	Measurements	Key Findings	Limitations	Related to Digital Literacy
**Arighi (2021)**	Quantitative descriptive	Italy, 108 patients with cognitive impairment	To describe the digital divide of a population of patients with dementia contacted by telemedicine during Italian lockdown for COVID-19 pandemic	Mini-mental status exam, whether patients were able to connect with neurologist on Microsoft Teams	Seventy-four patients connected with neurologist (successful televisit, 68.5%) and 34 patients were not able to perform televisit and were contacted by phone (failed televisit, 31.5%). No significant differences were observed among the two groups concerning age, gender, and education, but the prevalence of successful televisit was higher in the presence of younger caregivers	Small number of participants and the lack of accurate sociodemographic and socioeconomic information of care-givers	Digital divide is any uneven distribution in Information and Communications Technologies between people.
**Arthanat (2019)**	Qualitative descriptive	USA, Caregivers of ADRD (N= 24)	To beta-test a novel socially assistive robot (SAR) with a cohort of ADRD caregivers and gather their perspectives on its potential integration in the home context.	Guiding interview questions were derived from the UTAUT.	Adoption of the SAR, as an identified theme, was subject to the SAR's navigability, care recipient engagement, adaptability, humanoid features, and interface design. In contrast, barriers leading to potential rejection were technological	Both sources of data, the focus group and follow-up interviews, were derived from a single	Describing digital divide as a risk of rejection on socially assisted robot adoption (SAR)

Author (Publication Year)	Study Designs	Setting, Participants	Objectives	Measurements	Key Findings	Limitations	Related to Digital Literacy
					complexity, system failure, exasperation of burden, and failure to address digital divide	cohort of caregivers.	
**Banbury (2019)**	Qualitative descriptive	Australia, 69 dementia caregivers (Mean age 63 years; SD= 13.54)	To explore the use of technology and its perceived effects across different settings and countries	Three sub-scales from the e-Health Literacy Questionnaire (eHLQ): 'Using technology to process health information', 'Ability to actively engage with digital services' and 'Motivated to engage with digital services'	Providing peer-support groups using telehealth may have the potential to develop self-sustaining peer networks for isolated caregivers of people with dementia	Small sample size and potential bias in recruiting participants who were digitally proficient.	None
**Chirico (2022)**	Qualitative descriptive	The UK, Italy, Australia and Poland 127 informal carers (mostly female, n = 98) and 15 people with dementia (mostly female,	To explore the use of technology and its perceived effects across different settings and countries	interview questions not available	Technology Kept Us Alive During COVID-19; Remote care was anything but easy; Perceived Technology Limitations	People with dementia were underrepresented compared to an adequate number of carers	Digital literacy is familiar with technology, know how to use or manipulate digital devices

Author (Publication Year)	Study Designs	Setting, Participants	Objectives	Measurements	Key Findings	Limitations	Related to Digital Literacy
		mean age = 69)					
**Daly-Lynn (2023)**	Qualitative descriptive	The UK. 22 people with dementia (20 female, 2 male) and 31 formal caregiver survey	To examine experiences of people with dementia and live in technology enriched supported care models	Survey and interviews (questions were focused on the general experience of living in a supported living environment and the technology within the environment	A lack of awareness about living alongside technology	A single country in the United Kingdom	To use the system when they need to
**Duggleby (2019)**	Mixed methods	Canada; 199 carers of people with ADRD	To (1) examine differences at three months in the outcomes of hope, self-efficacy, and health-related quality of life (HRQOL) scores in users (ie, those who used MT4C at least once during the three-month period) compared with nonusers and (2) identify reasons for nonuse	Interviews,<br><br>Survey: Hope (Herth Hope Index; HHI), self-efficacy (General Self-Efficacy Scale; GSES), and HRQOL (Short-Form 12-item health survey version 2; SF-12v2)	Users had significantly higher GSES scores than nonusers (*P*=.048). Reasons for nonuse of MT4C included the following: caregiving demands, problems accessing MT4C (poor connectivity, computer literacy, and navigation of MT4C), and preferences (for paper format or face-to-face interaction). Problems accessing MT4C were related to poor internet connections, computer	Nature of a secondary analysis, low computer literacy and poor connectivity were not considered as exclusion criteria for the study, possible bias	Computer literacy is navigating

Author (Publication Year)	Study Designs	Setting, Participants	Objectives	Measurements	Key Findings	Limitations	Related to Digital Literacy
					literacy, and difficulties navigating the site.		
**Dupont (2023)**	Qualitative descriptive	Belgium; 18 family caregivers of people with dementia and 17 healthcare professionals	To define the content of an interactive website for people with dementia and their family caregivers to support them in ACP and to assess the barriers and facilitators for potential users in find- ing and using such a website from the perspective of family caregivers and healthcare professionals	Interviews: Preferences regarding functionalities – how the content is delivered (e.g., video), options for a larger font, text-to-speech option, etc. – and the possible barriers and facilitators to finding and using the website were assessed using open questions. Moreover, we also asked about the need for separate sections within the ACP website for people with dementia, family caregivers and dyads	Users had significantly higher General Self-Efficacy Scale scores than non-users. Web-based interventions, such as MT4C, have the potential to increase the self-efficacy of carers of persons with ADRD and MCC	As a nature of secondary analysis, thus, follow-up interviews with nonuser participants were not conducted.	Computer literacy refers to the ability to use computers and related technology efficiently
**Efthymiou (2022)**	Quantitative non-randomized	Cyprus and Greece (174 primary informal	To identify the levels of HL and eHL among carers of PwD in Greece and Cyprus and to search for the	eHEALS and health literacy (HL) survey	Primary informal carers reported a high level of eHealth Literacy (eHL) and HL. Carers with higher HL were more likely to report higher	A difficult sample to recruit, could not easily approach the	N/A

Author (Publication Year)	Study Designs	Setting, Participants	Objectives	Measurements	Key Findings	Limitations	Related to Digital Literacy
		carers of people with dementia	associations with other caring concepts		score of eHL, caregiving self-efficacy and lower score of problematic/dysfunctional coping. a positive message was received with regard to the role of HL and eHL in the everyday caring	carers who did not attend training and awareness events or day services.	
**Engelsma (2022)**	Qualitative (Delphi study)	The Netherlands: 37 ADRD experts (included 7~9 informal caregivers) age range between 21-70	To prioritize these through a Delphi study with ADRD experts (case managers, informal caregivers, hospital healthcare professionals, district nurses, and researchers)	Total three rounds: 1st round-participant characteristics and potentially new insights into barriers to mHealth use for older adults living with ADRD; 2nd round- consensus questionnaire- barriers to mHealth use for this population (its impact and frequency); 3rd round- rejudged those barriers for which no consensus or minor consensus	Twenty-six barriers are considered to majorly affect mHealth use, most of which relate to cognition and frame of mind. This study contributes to the development of mHealth design guidelines that take into account the progressive and diverse ADRD- and aging-related symptoms negatively affecting mHealth implementation and adoption	Passive recruitment of participants for this study proved to be challenging; the participant group may not be fully representative for all ADRD experts	Computer literacy
**Fischer (2024)**	Mixed methods	Brazile and India; 59 People with mild to	To explore the feasibility and acceptability of online or virtual CST (vCST) delivery in India	Interviews (intervention acceptability, feasibility, and experiences of implementation): surveys (caregivers- the Zarit Burden Interview,	While online services broadened geographic access, challenges emerged concerning inadequate computer literacy, poor technology	Not representative of the broader population of people	Computer literacy

Author (Publication Year)	Study Designs	Setting, Participants	Objectives	Measurements	Key Findings	Limitations	Related to Digital Literacy
		moderate dementia, supported by their family caregivers	and Brazil, emphasizing barriers and facilitators to implementation.	dementia caregiver experience scale)	access, and establishing interpersonal connections online. Exploratory, uncontrolled analyses indicated positive trends in quality of life but negative trends in cognition and activities of daily living, but these results were not statistically significant.	with dementia and their caregivers	
**Hicks (2023)**	Qualitative descriptive	The UK; 42 caregivers of people with dementia (20 co-resident and 22 non-co-resident carers)	To explore co-resident and non-co resident family carers of PLWD engaged with digital technologies during the pandemic	Interviews: (i) what were the participants' experiences and perceptions of the pandemic currently, (ii) how did these compare to the start of the lock-down (March 2020), and (Friedman et al.) what were their thoughts on how to move forward through the pandemic	Many of the carers engaged with Information and Communication Technologies, and to a lesser extent Assistive Technologies, during the pandemic	All carers were already participating within the wider DETERMIND study and so represent a population that may be more inclined to undertake research	Technology literacy
**Jakobssona (2022)**	Quantitative descriptive	Sweden; 32 participants (65-	To compare how older adults with cognitive impairment	The Short Everyday Technology Use	Interchangeably used with ICT literacy- is using digital technology, communications tools	Small sample size	none

Author (Publication Year)	Study Designs	Setting, Participants	Objectives	Measurements	Key Findings	Limitations	Related to Digital Literacy
		85 years old) with cognitive impairment of different origins	perceive relevance and level of Everyday Information and Communication Technologies (EICT)	Questionnaire (S-ETUQ)	and / or networks to access, manage, integrate, evaluate and create information in order to function in a knowledge society		
**Kotwal (2021)**	Quantitative descriptive study	USA; 20 dyads of caregivers and older adults with ADRD (mean age 70, SD = 9.0), 45% Spanish - speaking, 60% limited health literacy	To determine the feasibility of the advance care planning program	Validated 15-item ACP Engagement Survey, self-rated overall health; one validated health literacy question (ie, confidence with forms); confidence using the internet	15% participant felt comfortable using the internet. Surrogate supporting the patient with facilitating technology. ACP engagement scores increased for 16 of 20 (80%) patients (P=0.03) and 16 of 20 (80%) caregivers (P=0.18). Caregivers experienced increased knowledge (3.8 to 4.7, P = 0.002) and self-efficacy (3.6 to 4.5, P = 0.034) for ACP	Small sample size	Technological knowledge on computer and navigating the website
**McLoughlin (2023)**	Mixed methods	The UK, 39 carers of people with dementia	To explore the experiences of carers of people living with dementia who participated in	Online questionnaire (demographic status, and the type of support group that they attended by asking what happened at a typical	Themes (1) Perceptions of online support groups, and (2) Preferences for future support.	None	N/A

Author (Publication Year)	Study Designs	Setting, Participants	Objectives	Measurements	Key Findings	Limitations	Related to Digital Literacy
			videoconferencing support groups during the COVID-19 pandemic to investigate their preferences and experiences with online, hybrid, and face-to-face support.	session) and interviews (to elicit a more in-depth discussion of the participant's experiences with peer support groups, how the pandemic affected them as a carer, and what support they would like to receive in the future)			
**Oh (2016)**	Quantitative descriptive	USA; 204 Family and unpaid caregivers of people with ADRD	To investigate both chronic health conditions and the utilization of patient portals, focusing particularly on caregivers responsible for individuals with ADRD.	Survey (the Health Information National Trends Survey 2018–2020)	A significant proportion (46.6%) of ADRD caregivers had never accessed their patient portals. The limited utilization of patient portals among caregivers responsible for individuals with ADRD, particularly those with lower education, advanced age, and few chronic conditions, becomes apparent due to challenges associated with digital literacy and discomfort with computers	The cross-sectional nature of the data collected in the national database limits the ability to assess cause and effect, and only allows for investigation of relationships and the strength of those relationships	N/A

Author (Publication Year)	Study Designs	Setting, Participants	Objectives	Measurements	Key Findings	Limitations	Related to Digital Literacy
**Peri (2023)**	Qualitative descriptive	New Zealand; 12 family carers of people with dementia	To explore the roles and experiences of carers in accessing virtual CST	Interviews: capture information about the participant's experience of supporting a people with dementia to attend vCST during the COVID-19 pandemic, and to elicit suggestions on improving the implementation of vCST in a home environment	Carers reported positive responses to vCST that provided their family member living with dementia with social contact and cognitive stimulation during lockdown	A small sample size	N/A
**Ruggiano (2019)**	Qualitative descriptive	USA; 36 caregivers of people with ADRD	To capture a more in-depth perspective on how ADRD caregivers perceive existing and potential of technology to improve ADRD caregiving and care	interviews: (a) In what ways do you use technologies for caregiving? (b) How might a [clinical assessment tool, medication management tool, list of links and resources, alert system that sends messages to the provider] feature be helpful/not helpful for daily caregiving? (c) What kinds of caregiving tasks do you have challenges with that technology could potentially be of help?	Thematic findings suggest a conceptual model for designing ADRD caregiver technologies. The findings suggest that eHealth and individual technologies may not fully meet the needs of caregivers as they navigate the larger systems within which they provide care. Findings highlight the need to develop technologies for caregivers that are effective, easy to use, and	A small sample size representative of the larger caregiver population; focus group interviews did not allow the research team to fully explore the research question with each individual participant in the study	Computer literacy-ease of use

Author (Publication Year)	Study Designs	Setting, Participants	Objectives	Measurements	Key Findings	Limitations	Related to Digital Literacy
					more widely disseminated – especially for caregivers from disadvantaged backgrounds.		
**Smith (2022)**	Qualitative descriptive	Canada; 30 stakeholders and 22 patient-family participant dyads	To explore: (1) receptivity to SHARING Choices; (2) perceived barriers or facilitators for implementing SHARING Choices; and (3) adaptations to support SHARING Choices implementation	Interviews: receptivity to SHARING Choices components across CFIR domains	Enablers of SHARING Choices included adaptability of the intervention, purposive engagement of family (particularly for people with dementia), consistency with organizational priorities, and the relative advantage of SHARING Choices compared to current practices. Perceived barriers to implementation included intervention complexity, space constraints, workflow, and ACP hesitancy. The ACP facilitator was perceived as supportive in addressing individual and organizational implementation barriers including	A small sample size; limited racial and ethnic diversity of the participants, small heterogenous group	N/A

Author (Publication Year)	Study Designs	Setting, Participants	Objectives	Measurements	Key Findings	Limitations	Related to Digital Literacy
					patient health and technology literacy and clinician time for ACP discussions		
**Thompson (2024)**	Quantitative randomized controlled trial	USA; 163 family caregivers of persons with dementia	To evaluate the feasibility and usability of a technology-delivered intervention designed for family caregivers	Computer Proficiency was measured by the Computer Proficiency Questionnaire short form (CPQ-12)	CPQ-12 scores can be used as a screening tool to identify those who may need additional support to engage with and benefit from technology-delivered interventions.	This sample was highly educated and proficient with technology.	Computer proficiency
**Wang (2021)**	Quantitative non-randomized	China; 300 primary family caregiver - care recipient dyads	To examine the association between eHealth literacy, education, and caregiver burden among Chinese caregivers of older adults with cognitive impairment	Eight-item Chinese eHealth Literacy Scale	An interaction effect between eHealth literacy and education on caregiver burden was identified. eHealth literacy was positively associated with caregiver burden among caregivers with less than a high school education, but not among those with a high school education or above. eHealth literacy is salient in the burden experienced by caregivers with low education	cross-sectional in nature	eHealth literacy

Author (Publication Year)	Study Designs	Setting, Participants	Objectives	Measurements	Key Findings	Limitations	Related to Digital Literacy
**Wilding (2021)**	Qualitative descriptive	Australia; 39 participants (carers, volunteer, healthcare staffs of persons with dementia, average age was 66 years old) and mostly female (82%)	To increase access to information, support, and connection for carers of rural people living with dementia, via a co-designed, integrated website/mobile application (app) and Zoom videoconferencing	Qualitative data (memos, transcripts of focus groups and interviews)	The volunteers reported that the Verily Connect app was easy to use and they felt they derived benefit from volunteering. The volunteers had less volunteering work than they desired due to low numbers of carer participants; they reported that older rural carers were partly reluctant to join the trial because they eschewed using online technologies, which was the reason for involving volunteers from each local community	Small sample size	N/A
**Yin (2024)**	Quantitative descriptive	USA; 140 caregivers of people with dementia	To investigate the perceptions and utilization of online peer support through a survey	Survey (eHeal)	Our findings show that the behavior of accessing any online community was significantly associated with participants' belief in the value of online peer support (P=.006).	Selection bias	eHealth literacy

Note. ADRD = Alzheimer's diseases and related diseases; CST = cognitive stimulation therapy; N/A = not applicable
